# Postpartum uptake of contraception in rural northern Malawi: A prospective study^☆^

**DOI:** 10.1016/j.contraception.2016.05.007

**Published:** 2016-11

**Authors:** Aisha N.Z. Dasgupta, Basia Zaba, Amelia C. Crampin

**Affiliations:** a London School of Hygiene and Tropical Medicine, Keppel Street, London, WC1E 7HT, UK; b Karonga Prevention Study, Chilumba, Karonga District, Malawi

**Keywords:** Contraception, Postpartum, Malawi, Postpartum abstinence, Amenorrhea

## Abstract

**Objectives:**

Cross-sectional estimates of contraceptive use do not provide understanding of time to postpartum uptake. This paper uses a range of Malawian data sources: a prospective study to explore time to uptake of contraception and a cross-sectional survey to assess whether sexually active postpartum women whose fecundity has returned use contraception, and whether abstaining/amenorrheic women report using contraception.

**Study design:**

A demographic surveillance site (DSS) in Malawi was used to identify 7393 women aged 15–49 years eligible for a 1-year prospective study of contraception using provider-recorded data on patient-held records (2012–2013). This provided a reliable record of time to uptake of postpartum contraception. The average timing of resumption of sexual activities after postpartum abstinence and return of menses was estimated from a population-based sexual behaviour survey in the DSS (2010–2011).

**Results:**

Of 4678 women recruited to the prospective contraception study, 442 delivered an infant during the observation period. Of these, 28.4% used modern contraception within 6 months of delivery. However, at 6–9 months after delivery, only 28.0% women had started menstruation and resumed sexual activities; of these, 77.6% used contraception. Amongst abstaining/amenorrheic women, a quarter reported contraceptive use.

**Conclusions:**

The low uptake of postpartum contraception is likely due to many women abstaining and/or experiencing amenorrhea. Self-reports of contraceptive use amongst abstaining/amenorrheic women bring into question the quality of cross-sectional surveys and demonstrate that contraceptive use by women at low risk of pregnancy could contribute to the Malawi paradox of high contraceptive use and high fertility. Given relatively low risk of pregnancy in the postpartum period in this context, a focus on long-acting/permanent methods may be more effective to avert unintended pregnancies.

**Implications:**

There has been increasing interest in the utility of postpartum contraceptive programmes to assist women to space births. Our findings suggest that, although uptake of contraception is low, this is partly due to postpartum abstinence and amenorrhea. Provision of long-acting/permanent methods will be more effective for women after delivery.

## 1 Introduction

Women who have recently given birth can be cost-effectively targeted for family planning (FP) education and engagement because often they are already connected with health professionals [1], [2], [3]. In Malawi, women are expected to contact health services either for themselves or their infant during the first 9 months after delivery. Although 42% of married women in Malawi reported using modern contraception in cross-sectional surveys in 2010 (higher than other countries in the region), the total fertility rate remains high at 5.7 [4]; this disconnect between contraceptive use and fertility deserves exploration. Maternal mortality is high, estimated at 675 deaths per 100,000 live births, and underfive mortality is estimated at 112 deaths per 1000 live births in 2010 [4]. Contraception to help women prevent mistimed or unintended pregnancies is an important public health intervention.

The postpartum period refers to the time from delivering a baby until menses start again, which varies between women. Some researchers refer to an immediate 6-week postpartum period for clinical reasons or an extended postpartum period that is the first year after birth [5], [6]. This 1-year postpartum period is important for the welfare of mother and child and is complex in terms of fertility return: sexual activity, breastfeeding, amenorrhea, postpartum reduced fecundity and use of contraception may all change rapidly [7], [8]. Typically, postpartum women do not wish to become pregnant again soon after delivering an infant, but many women in sub-Saharan Africa do not use contraception during this time [5]. Sexually active women whose fecundity has returned are at risk of unintended pregnancy.

Postpartum abstinence (PPA) in Malawi is a traditional means to space births, and in northern Malawi, traditions are strengthened because women are considered to be “polluted” after delivering a baby until menses have returned [9]. A study found that northern Malawi has longer PPA than other regions (17 months in northern Malawi compared to 10 months in the south and 7 months in the central region, in 1998) [10]. However, it appears that the duration of PPA in Malawi has been decreasing in the context of the HIV epidemic, as women may be concerned that their husbands would stray [11]. A more recent estimate by the Demographic and Health Survey (DHS) using cross-sectional data estimates the median duration of PPA to be 4.6 months in Malawi overall, 4.9 months for the northern region, 3.1 in the central region and 6.5 in the southern region [4].

Breastfeeding lengthens postpartum amenorrhea and reduces the risk of pregnancy [12], but if breastfeeding is not exclusive, then the protective benefits decline. Although 99% of 4–5 month olds are currently breastfeeding, only 40% are exclusively breastfed. The median duration of amenorrhoea is 10.5 months [4]. Time to uptake of contraception needs to be considered in the context of PPA and amenorrhea.

## 2 Study setting and methods

Cross-sectional estimates of contraceptive use do not provide understanding of time to postpartum uptake. The DHS developed contraceptive calendars, which retrospectively record women's contraceptive method, pregnancies, births, breastfeeding and pregnancy terminations every calendar month for the 5 years prior to interview. These data are subject to memory recall issues and are unable to examine prospective time to uptake of contraception, which requires different methods. We draw on a range of data sources. We use prospective data to more reliably capture time to uptake of postpartum contraception and cross-sectional data to explore whether sexually active postpartum women whose fecundity has returned are using contraception and whether abstaining/amenorrheic women report using contraception.

### 2.1 Study setting

The Karonga Prevention Study (KPS) operates a demographic surveillance site (DSS) in northern rural Malawi, where all births, deaths and migrations are recorded. At the end of 2012, 36,524 individuals were under observation [13]. For individuals who participate in multiple KPS studies, it is possible to link data using unique identifiers, providing the opportunity to draw on information from a range of sources.

A range of contraceptive methods are provided in the area through different mechanisms (public, private, clinic and outreach) and service providers (Clinical Officers, Nurses, Medical Assistants, Health Surveillance Assistants and Community-Based Distribution Agents). Both KPS (2008–2009) and DHS (2010) estimate the contraceptive prevalence at 45% (including condoms) in Karonga using cross-sectional data [4], [14].

### 2.2 Prospective FP data

FP cards were offered to all women aged 15–49 years living in the KPS DSS between January and April 2012 by key informants. The FP card was attached to the woman's health passport (paper medical record) and the woman was asked to keep her FP card for 1 year. All 132 health care providers working in the study catchment area were trained to record information on the FP cards whenever they provided contraception to a woman holding a card. Multiple refresher trainings were conducted, mobile phone air time was provided and motivational text messages were sent to health care providers, to keep them engaged [15].

After 1 year, the field team collected the FP cards and used health passports and questioning to complete any missing contraceptive episodes. The majority (87%) of data were collected in the prospective format (in the form of written reports by service providers) but gaps in data were filled retrospectively (from the women's reports), achieving a more complete and accurate longitudinal dataset than would be possible using conventional survey methods. The field team took informed written consent from study participants upon collection of the FP cards. Consent was not requested at the beginning of the study because the key informants were not members of the KPS study staff and could not administer the informed consent procedure. Ethical approval was granted by the College of Medicine Research and Ethics Committee, Malawi, and the ethics committee at the London School of Hygiene and Tropical Medicine, UK.

### 2.3 Explanatory variables

Explanatory variables for uptake of postpartum contraception by women participating in the prospective FP study were derived and linked from the DSS database (age and distance to road) or from past KPS population-based HIV serosurveys, socioeconomic surveys and adult sexual behaviour surveys. These surveys contributed information on HIV, educational level, marital status and prospective fertility intentions. The time-varying covariates (e.g. HIV, marital status and fertility intentions) refer to the status provided most recently when the woman received the FP card and were considered unknown if recorded more than 2 years prior to receiving the card.

### 2.4 Risk and exposure to pregnancy

A separate analysis (not linked to the prospective FP study) provided an understanding of risk and exposure to pregnancy amongst postpartum women in Karonga. The proportion of women who resumed sexual activities and started menstruation after childbirth was estimated using data from the last round of the KPS adult sexual behaviour survey amongst women aged 15–49 years in 2010–2011, using the questions “Have you had sex in the last month?” and “When was your last period?”. This was analysed with a cross-sectional self-report on modern contraception use from the same survey. These cross-sectional data were not capable of estimating time to uptake of contraception, which is why the prospective FP dataset is so useful. It was assumed the woman had not had sex since delivery if she reported no sex in the last month. If a woman responded positively that she had sex in the last month, it was known that she had resumed sexual activity, although the date of resumption was unknown. It was not possible to estimate the average length of PPA using these data, as the survey was not designed for this purpose.

### 2.5 Analysis methods

Using birth data from the DSS, the length of birth interval was calculated for women who delivered two or more children in the DSS area. The prospective FP data were linked to the KPS database, and Stata 12 (StataCorp 2011 LP, College Station, TX, USA) was used to analyse the data. Analysis was restricted to women who delivered a baby (as recorded in the DSS database) after she received the FP card. Survival analysis was used to examine time to uptake of female-controlled contraception following delivery. Ever-use (during the study year) of condoms was recorded, but episodes of condom use were not identified individually, so this analysis focuses on uptake of modern contraception excluding condoms.

## 3 Results

### 3.1 DSS birth intervals

**Fig. 1:**
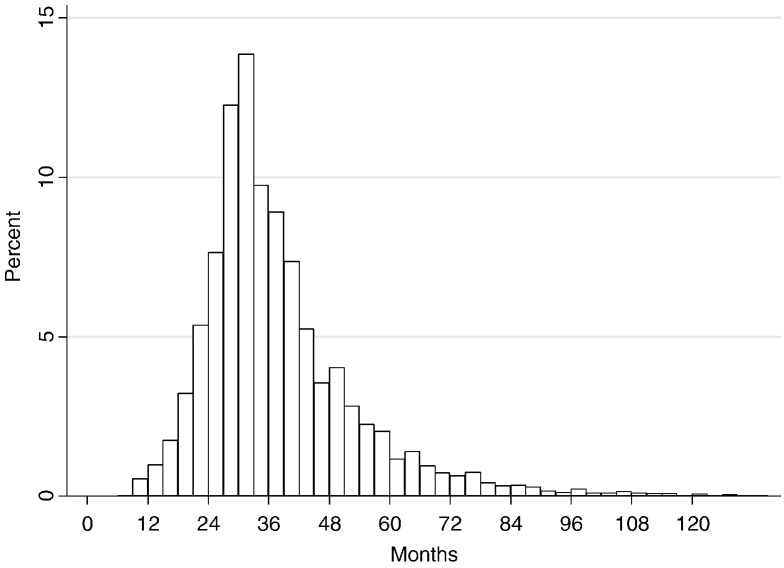
Time between delivery of child and birth of the next child.

The median birth interval is 37 months, and 11% of births are estimated to have occurred less than 24 months after the previous birth (Fig. 1). It was estimated that 92% of deliveries to women in the DSS in 2010 took place in a health facility rather than at home, and 13.9% took place in the district hospital in Karonga town, demonstrating a relatively high level of institutional deliveries.

### 3.2 Prospective FP study participants

Of 7393 women aged 15–49 years eligible to participate in the FP study, 4678 (63.3%) both received and submitted an FP card. Of these, 442 women (9.4%) delivered a baby after receiving their FP card.

The average observation time from delivering a baby to exiting the study was 270 days. There were 253 person-years time at risk. No woman delivered more than once during the observation period. Of 169 women who used contraception, Table 1 presents the first contraceptive method that was used after delivery. The majority of postpartum contraceptive users initiated with injectables (59.8%). The method mix and contraceptive prevalence using DHS Karonga District data are presented for background.

### 3.3 Time to uptake of contraception

**Fig. 2:**
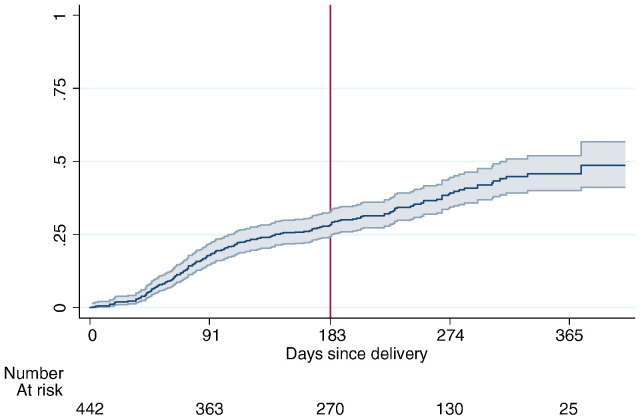
Probability of postpartum uptake of contraception.

Fig. 2 shows the Kaplan–Meier estimated “failure” curve (with 95% confidence interval) from time of delivering a baby to uptake of contraception. By 6 months, 28.4% of women had used modern contraception. By 365 days, 45.8% had used modern contraception. A handful of tubal ligations and implant procedures were performed within the “immediate” postpartum period: three tubal ligations were performed within 1 week of delivering a baby, one tubal ligation was performed at 3 weeks and two implants were inserted at 2 weeks and 5 weeks, respectively.

### 3.4 Profile of postpartum contraceptive users

Women who successfully managed to use a modern method of contraception within 6 months were compared to women who did not (Table 2). Married women, women who attained higher levels of education and those living within 1 km of a road were more likely to use contraception within 6 months, although this failed to reach statistical significance.

### 3.5 Method switching

Of the 169 women who did use a modern method of contraception at any time after delivering, just five used more than one method during the course of the observation period: three started with injectables and switched to an implant and two used both injectables and oral contraceptive pills.

### 3.6 PPA and amenorrhea

Presumably not all women were sexually active and had commenced menstruation and hence would not need contraception. Regardless of the reasons for resumption (or not) of sexual activities and bearing in mind the culture of PPA, use of postpartum contraception was analysed against a denominator of postpartum women who were sexually active and had started menstruation.

A separate analysis used data for 872 women in the sexual behaviour survey who delivered a baby between 0 and 365 days prior to interview. Table 3 shows that the proportion of women who had sex in the last month and commenced menstruation increased steadily with time since delivery, although even between 6 and 9 months, only a quarter of women were both sexually active and periods had returned (28.0%). Across the four time periods since delivery, amongst women whose menses had returned and had sex in the last month, around three quarters reported using modern contraception (63.4–77.6%). Amongst women who had not had sex in the last month, 26.8% (124/462) reported currently using contraception (of which 38% were using injectables, 37% were using condoms and 11% were using tubal ligations). Amongst women who had not had a period since birth, 29.3% (151/515) reported using contraception (of which 27% were using injectables, 49% were using condoms and 8% were using tubal ligations) (data not shown).

## 4 Discussion

At first glance, the prospective FP data revealed that there was relatively poor uptake of contraception after childbirth. This is despite women in antenatal, postpartum and vaccination clinics receiving intensive education messages on birth spacing. However, on further investigation, data from the cross-sectional adult sexual behaviour survey indicated that a significant proportion of women in the first year since delivery were not yet sexually active and/or had not commenced menstruation, suggesting that they were not at high risk of pregnancy. Of those who were sexually active and menses had returned, around three quarters reported modern contraceptive use, which is high.

Amongst women who were either abstinent or had not started their periods, around a quarter reported using contraception. This could be interpreted in two ways: (a) overreporting of contraceptive use in cross-sectional surveys (for abstinent women reporting use of condoms, this is not accurate: if they had not had sex in the last month, they cannot be currently using condoms) and (b) use of contraception by women who are at low risk of pregnancy. Both could explain the Malawi paradox of apparently high contraceptive use and high fertility.

Elsewhere we show high discontinuation of short-term methods in northern Malawi [16]. Therefore, provision of short-term methods in the immediate postpartum period is unlikely to be effective, given the low risk of pregnancy for women during this time and high probability of discontinuation by the time the woman resumes sexual activity and menstruation [17]. According to our prospective FP study, only a handful of women received a long-acting or permanent method of contraception within weeks of delivering their baby. Given that a large proportion of deliveries now take place at health facilities in this area (92%), this provides an opportunity for health care providers to either counsel women on their FP options before they return home (perhaps providing a date by when the woman should return to the health facility to start contraception) or indeed to provide a service such as an implant or intrauterine device (IUD) insertion [18], [19], [20], [21].

There is good evidence that longer birth intervals have beneficial health outcomes for mother and child [22], [23], [24], [25]. It was estimated that 11% of births occurred less than 24 months apart. For comparison, the Malawi DHS estimates the median birth interval to be 36 months and 15% of births to occur less than 24 months apart [4], which is in line with estimates from Tanzania, Zambia and Mozambique, but otherwise relatively low compared to other countries in the region [26], demonstrating that the majority of children were relatively well spaced. Therefore, services should be strengthened to encourage the few women who are at risk of experiencing birth intervals of less than 24 months to use contraception, and they would need to be identified beforehand.

Measuring contractive uptake during the extended postpartum period is challenging, and the prospective data collected in this setting were a strength. Limitations to the prospective FP study include limited follow-up time due to restrictions in time and resources, which is problematic given lingering protective factors (amenorrhea, abstinence), and potential biases introduced from asking women to self-report on contraceptive use. Male-controlled methods of contraception were underestimated using this method of data collection. Relying on cross-sectional data, we had assumed that women had not had sex at any time since delivery if she reported no sex in the last month in the adult behaviour survey, and this was a limitation and ultimately underestimated the proportion of women who had resumed sexual activities. Conception risks in the first 6 months postpartum are small for women experiencing amenorrhea, regardless of whether or not they are exclusively breastfeeding. Nevertheless, there is still a risk of pregnancy prior to return of menses.

In conclusion, use of contraception in the immediate postpartum period by women at low risk of pregnancy could be a contributing factor to the Malawi paradox of high contraceptive use and high fertility. Promotion of long-acting and permanent methods of contraception for those women who want to space or stop their births is key.

## Figures and Tables

**Table 1:** Method mix for women who started using contraception after delivering a baby within study period, compared to DHS figures (excluding condoms)

Method	Number of women who started the method	Method mix (%) for postpartum contraceptive users	Method mix (%) DHS, Karonga District^a^	Contraceptive prevalence, DHS, Karonga District^a^ (%)
Tubal ligation	12	7.1	33.2	12.1
Implant	35	20.7	8.8	3.2
IUD	0	0.0	0.0	0.0
Injectables	101	59.8	54.5	19.9
Oral contraceptive pill	21	12.4	3.6	1.3
**Total**	**169**	**100.0**	**100.0**	**36.5**

a According to cross-sectional data for Karonga District, DHS, for comparison. Note that this population is married women contraceptive users, not restricted to postpartum women.

**Table 2:** Profile of women who started contraception within 6 months compared to women who did not start using contraception within 6 months (excludes women who were not observed for at least 6 months)

	Started contraception within 6 months		Did not start contraception within 6 months		Total	χ^2^ test^a^
Mean age	26.0		26.7		26.5	p = .27^b^
	*N*	%	*N*	%	*N* (%)	
**Marital status**						p = .08
Currently married	117	32.7	241	67.3	358 (100%)	
Separated/widowed/divorced	2	10.5	17	89.5	19 (100%)	
Never married	3	20.0	12	80.0	15 (100%)	
**Education**						p = .09
Incomplete primary	3	13.0	20	87.0	23 (100%)	
Complete primary	70	30.3	161	69.7	231 (100%)	
Secondary +	49	35.5	89	64.5	138 (100%)	
**HIV status**						p = .71
HIV positive	4	26.7	11	73.3	15 (100%)	
HIV negative	109	31.2	240	68.8	349 (100%)	
**Parity**						p = .64
None	4	30.8	9	69.2	13 (100%)	
1–4	73	29.8	172	70.2	245 (100%)	
5 +	23	35.9	41	64.1	64 (100%)	
**Fertility intention**						p = .05
No more	15	26.8	41	73.2	56 (100%)	
Wait 2 + years	51	38.1	83	61.9	134 (100%)	
Want within 2 years	19	21.1	71	78.9	90 (100%)	
Unsure	5	23.8	16	76.2	21 (100%)	
**Distance to road**						p = .16
Less than 1 km	60	35.1	111	64.9	171 (100%)	
More than 1 km	63	28.4	159	71.6	222 (100%)	

a p Values from χ^2^ testing difference between “did start contraception within 6 months” and “did not start contraception within 6 months of delivering”.

b Two-sample *t* test, testing difference between means.

**Table 3:** Women who have resumed sexual activities and menses had returned and proportion of women who are using modern contraception, by time since delivery

Time since delivery	Number of women	Women who had sex in last month and had a period since delivery, *N* (%)	Of those who had sex in the last month and periods had returned, women who self-report they are currently using contraception, *N* (%)
0–3 months	199	12 (6.0%)	9 (75.0%)
3–6 months	226	41 (18.1%)	26 (63.4%)
6–9 months	207	58 (28.0%)	45 (77.6%)
9–12 months	222	79 (35.6%)	55 (69.9%)
